# Predictors of short-term thrombocytopenia after transcatheter aortic valve implantation: a retrospective study at a single Japanese center

**DOI:** 10.1186/s13104-020-05386-7

**Published:** 2020-11-16

**Authors:** Yasutaka Yamada, Daisuke Miura, Ayako Takamori, Eijiro Nogami, Junji Yunoki, Yoshiro Sakaguchi

**Affiliations:** 1 Department of Anesthesiology and Critical Care Medicine, Faculty of Medicine, Saga University, 5-1-1 Nabeshima, Saga City, Saga 849-8501 Japan; 2 Department of Anesthesia, Saga Medical Center KOSEIKAN, Saga City, Saga Japan; 3 Clinical Research Center, Saga University Hospital, Saga City, Saga Japan; 4 Department of Thoracic and Cardiovascular Surgery, Saga Medical Hospital, Saga City, Saga Japan

**Keywords:** Transcatheter aortic valve implantation, Aortic stenosis, Thrombocytopenia, Platelet count, Balloon-expandable valve

## Abstract

**Objective:**

Thrombocytopenia is common after transcatheter aortic valve implantation (TAVI) and is associated with mortality and major complications, although the underlying mechanisms are unclear. This retrospective single-center study aimed to identify factors associated with the decrease in platelet count (DPC) after TAVI in Japanese patients. Patients with severe aortic valve stenosis who underwent transfemoral TAVI between March 2014 and August 2019 were grouped according to DPC values of < 50% or ≥ 50% (DPC = 100% × [baseline platelet count–nadir platelet count]/[baseline platelet count]). Multivariable logistic regression analysis was performed to identify factors associated with a DPC of ≥ 50%.

**Results:**

Among the 131 patients who underwent transfemoral TAVI, 74 patients (56%) had a DPC of ≥ 50%, and 57 patients (44%) had a DPC of < 50%. Significant risk factors for a DPC of ≥ 50% were older age, lower body mass index (BMI), and use of balloon-expandable valves (BEV). The multivariable analysis revealed that a DPC of ≥ 50% was independently predicted by low BMI (adjusted odds ratio: 0.884, 95% confidence interval: 0.785–0.997; *P *= 0.039) and BEV use (adjusted odds ratio: 3.014, 95% confidence interval: 1.003–9.056; *P *= 0.045). Platelet count monitoring after TAVI, especially when using BEV devices, is essential for Japanese patients with low BMI.

## Introduction

Transcatheter aortic valve implantation (TAVI) is used to treat high-risk patients with severe aortic stenosis [1–3]. However, TAVI-related thrombocytopenia is common [4–18], with platelet counts decreasing to < 100 × 10^3^/μL in more than one-third of patients who undergo TAVI [4, 5]. Previous studies have shown that the nadir platelet count is approximately 50–60% of the baseline value, occurs on postoperative day 2–3, and begins to recover after postoperative day 5 [4, 6–9]. Thrombocytopenia after TAVI is associated with poor clinical outcomes [4, 8–12], and Dvir et al. have reported that patients with a large decrease in platelet count (DPC, ≥ 50%) had lower 1-year survival rates than patients with a smaller decrease in DPC (1-year survival: 65.8% vs. 83.9%, *P *< 0.001) [4]. The mechanisms underlying thrombocytopenia after TAVI are unclear and likely multifactorial, which may involve enhanced platelet consumption, reduced platelet production, and/or significant haemodilution [9]. Other studies have suggested that post-TAVI thrombocytopenia is predicted by the use of balloon-expandable valves (BEVs) [7, 8, 13], contrast medium volume [5, 10], coronary artery disease [8], left ventricular ejection fraction [8], blood transfusions [13, 14], and a smaller aortic valve area [15]. Lower body mass index (BMI) may also be associated with an increased risk of post-TAVI thrombocytopenia [15]; though this relationship remains controversial, as previous studies have generally evaluated European or American patient populations [4–8, 10, 12, 14–16], which have larger physical statures. This retrospective study aimed to identify factors that might predict post-TAVI thrombocytopenia in Japanese patients, who are more likely to have smaller physical statures.

## Main text

### Methods

#### Study population

This retrospective study included patients who underwent transfemoral TAVI for severe aortic valve stenosis at our institution between March 2014 and August 2019. We excluded patients who underwent transapical or trans-subclavian TAVI. The retrospective study protocol complied with the Declaration of Helsinki and was approved by the institutional review board of the Faculty of Medicine, Saga University (20190703, September 30, 2019), which waived the requirement for informed consent. Study details were published on the institution’s website and patients were allowed to opt-out of the research use of their data.

#### TAVI procedures

The TAVI procedures were performed in a hybrid operating room under general anaesthesia, with TAVI access and valve size selected based on three-dimensional computed tomography measurements. Transarterial access was established percutaneously or after cut-down in a standard manner. Decisions regarding vascular access, valve type, and valve size were made by the institution’s cardiovascular team. Patients were treated using either Edwards Sapien XT or Sapien 3 BEVs (Edwards Lifesciences, Irvine, CA, USA), or Medtronic CoreValve, EvoluteR or EvolutePro self-expanding valves (SEVs; Medtronic, Minneapolis, MN, USA). Rapid right ventricular pacing was performed during balloon dilation for a native aortic valve and at the time of BEV implantation. We used transoesophageal echocardiography to confirm appropriate valve positioning and identify any para-valvular leak, which prompted balloon post-dilation, if necessary. All patients received unfractionated heparin to maintain a minimum activated clotting time of > 250 s during the procedure. Protamine (1 mg for each 100 U heparin) was routinely administered at the time of vascular closure, which was performed in a standard manner. The contrast agent was iopamidol, which is iodinated, non-ionic, and has low osmolarity. The tracheal tube was removed in the operating room if normal results were observed for respiratory status and circulatory dynamics. All patients were admitted to the intensive care unit after surgery.

#### Definitions and data collection

Baseline parameters were selected based on the last evaluation before the procedure. Variables included age, sex, BMI, hypertension, diabetes mellitus, cancer, peripheral artery disease, cerebrovascular disease, chronic kidney disease, known coronary artery disease, atrial fibrillation, pacemaker implantation, Society of Thoracic Surgeons Predictive Risk of Mortality score, logistic EuroSCORE, echocardiographic findings, valve type (BEV or SEV), contrast medium volume, operating time, intraoperative blood loss, blood transfusion, percutaneous cardiopulmonary support (PCPS), balloon post-dilation, and laboratory parameters. Procedural events were defined according to the Valve Academic Research Consortium-2 criteria [19].

Platelet counts were measured using a Sysmex XN-900 system (JACLaS, Tokyo, Japan) before TAVI, day 0, day 1, day 2, day 3, day 5, day 7, and then as needed. The nadir platelet count was defined as the lowest platelet count up to 14 days after TAVI. The DPC was calculated as 100% × (baseline platelet count − nadir platelet count)/(baseline platelet count), and the results were categorised as a DPC of ≥ 50% or < 50%. Clinicodemographic characteristics, intraoperative findings, and clinical outcomes were compared between the groups with DPCs of ≥ 50% and < 50%.

#### Statistical analysis

Categorical variables were reported as number (percentage) and compared using the Chi-squared test or Fisher’s exact test. Continuous variables were reported as median (interquartile range [IQR] and range) and compared using Student’s *t*-test or the Mann–Whitney *U* test. Multivariate logistic regression analysis was performed to identify factors that might predict a DPC of ≥ 50%. Potentially relevant factors were selected from variables with a univariate *P*-value < 0.05 and previously reported factors [5, 8, 10, 13–15]: coronary artery disease, left ventricular ejection fraction, aortic valve area, contrast medium volume, and blood transfusion. The results were reported as odds ratios (OR) and 95% confidence intervals (CIs). Significant differences were identified using two-sided *P*-values of < 0.05. All analyses were performed using JMP Pro software (version 13; SAS Institute Inc., Cary, NC, USA).

### Results

Between March 2014 and August 2019, 145 patients underwent TAVI; 14 patients who underwent transapical or trans-subclavian TAVI were excluded. Thus, the analyses included 131 patients who underwent transfemoral TAVI. The median preoperative platelet count was 175 × 10^9^/L (IQR: 143–215 × 10^9^/L, range: 60–426 × 10^9^/L) and the median nadir platelet count was 82 × 10^9^/L (IQR: 62–108 × 10^9^/L, range: 26–256 × 10^9^/L) (Additional file 1: Figure S1). The median time to the nadir platelet count was postoperative day 3 (Additional file 2: Figure S2) and the median DPC was 51% (DPC of ≥ 50%: 74 patients, DPC of < 50%: 57 patients). No patients required platelet transfusions during the perioperative period.

The baseline characteristics of the patients according to DPC categorisation are shown in Table 1.

**Table 1:** Baseline characteristics of the study population

	Total (n = 131)	DPC of ≥ 50% (n = 74)	DPC of < 50% (n = 57)	*P*-value
Age (years)	84.5 ± 4.7	85.3 ± 3.9	83.5 ± 5.4	0.031
Men	44 (33.6%)	22 (29.7%)	22 (38.6%)	0.287
Body mass index (kg/m^2^)	22.3 ± 3.4	21.7 ± 3.2	23.1 ± 3.6	0.016
Hypertension	107 (81.7%)	61 (82.4%)	46 (80.7%)	0.799
Diabetes mellitus	33 (25.2%)	16 (21.6%)	17 (29.8%)	0.284
Cancer	17 (13.0%)	10 (13.5%)	7 (12.3%)	0.835
Peripheral arterial disease	14 (10.7%)	8 (10.8%)	6 (10.5%)	0.958
Cerebrovascular disease	22 (16.8%)	16 (21.6%)	6 (10.5%)	0.092
Chronic kidney disease	99 (75.6%)	55 (74.3%)	44 (77.2%)	0.705
Coronary artery disease	70 (53.4%)	41 (55.4%)	29 (50.9%)	0.607
Atrial fibrillation	36 (27.5%)	17 (22.9%)	19 (33.3%)	0.188
Pacemaker implantation	8 (6.1%)	6 (8.1%)	2 (3.5%)	0.276
STS (%)	6.8 ± 3.7	7.0 ± 3.7	6.4 ± 3.8	0.382
Euro Score (%)	18.1 ± 10.8	19.3 ± 11.2	16.6 ± 10.2	0.163
EF (%)	65.0 ± 14.9	65.4 ± 15.2	64.6 ± 14.7	0.763
AVA (cm^2^)	0.63 ± 0.17	0.62 ± 0.16	0.64 ± 0.19	0.479
mPG (mm Hg)	50.3 ± 18.3	51.4 ± 17.9	48.8 ± 18.9	0.429

DPC: decrease in platelet count; STS: Society of Thoracic Surgeons Predictive Risk of Mortality score; EF: ejection fraction; AVA: aortic valve area; mPG: mean pressure gradient

Data are presented as mean ± standard deviation or n (%)

Patients with a DPC of ≥ 50% were significantly older (85.3 years vs. 83.5 years, *P *= 0.031) and had a significantly lower BMI (21.7 kg/m^2^ vs. 23.1 kg/m^2^, *P *= 0.016). There were no significant inter-group differences in operation time, contrast medium volume, intraoperative blood loss, blood transfusion, use of PCPS, balloon post-dilation, or the various clinical outcomes (Table 2).

**Table 2:** Perioperative findings and clinical outcomes

	Total (n = 131)	DPC of ≥ 50% (n = 74)	DPC of < 50% (n = 57)	*P*-value
Valve type (BEV)	110 (84%)	67 (90.5%)	43 (75.4%)	0.019
Contrast medium volume (mL)	86.7 ± 26.0	83.8 ± 18.9	90.5 ± 32.7	0.144
Operating time (min)	116.1 ± 45.5	113.6 ± 45.8	119.3 ± 45.3	0.479
Intraoperative blood loss (mL)	84.6 ± 11.5	90.2 ± 139	77.2 ± 73.9	0.526
PCPS	15 (11.5%)	8 (10.3%)	7 (12.3%)	0.793
(prophylactic/emergency)	(13/2)	(6/2)	(7/0)	
Post-balloon dilation	39 (29.8%)	23 (31.1%)	16 (28.1%)	0.709
Intubation time of > 48 h	3 (2.3%)	2 (2.7%)	1 (1.8%)	0.730
Intensive care unit stay (days)	1.9 ± 4.6	1.6 ± 1.9	2.4 ± 6.7	0.328
Pacemaker implantation	10 (7.6%)	3 (4.1%)	7 (12.3%)	0.079
Atrial fibrillation	10 (7.6%)	5 (6.8%)	5 (8.8%)	0.667
Major stroke	2 (1.5%)	2 (2.7%)	0 (0%)	0.211
Acute kidney injury	3 (2.3%)	2 (2.7%)	1 (1.8%)	0.710
Tracheotomy	1 (0.7%)	0 (0%)	1 (1.8%)	0.253
Blood transfusions	64 (49%)	36 (48.7%)	28 (49%)	0.957
Infection	0 (0%)	0 (0%)	0 (0%)	–
Sepsis	0 (0%)	0 (0%)	0 (0%)	–
Death	0 (0%)	0 (0%)	0 (0%)	–

DPC: decrease in platelet count; BEV: balloon-expandable valve; PCPS: percutaneous cardiopulmonary support

Data are presented as mean ± standard deviation or n (%)

However, the group with a DPC of ≥ 50% was more likely to receive BEVs (90.5% vs. 75.4%, *P *= 0.019). Table 3 shows the results of the analyses of factors that predicted a DPC of ≥ 50% after TAVI.

**Table 3:** Univariable and multivariable analyses of factors associated with a DPC of ≥ 50%

	Unadjusted			Adjusted		
	Odds ratio	95% CI	*P*-value	Odds ratio	95% CI	*P*-value
Age (years)	1.088	1.006–1.177	0.029	1.078	0.981–1.185	0.105
Body mass index (kg/m^2^)	0.879	0.789–0.979	0.016	0.884	0.785–0.997	0.039
Valve type (BEV)	3.116	1.164–8.343	0.019	3.014	1.003–9.056	0.045
Coronary artery disease	1.247	0.532–2.924	0.611	1.593	0.592–4.296	0.351
EF (%)	1.004	0.981–1.027	0.761	1.016	0.988–1.045	0.255
AVA (cm^2^)	0.479	0.064–3.608	0.475	0.456	0.042–4.986	0.519
Contrast medium volume (mL)	0.989	0.976–1.004	0.141	0.991	0.975–1.008	0.273
Blood transfusions	0.981	0.492–1.958	0.957	0.897	0.395–2.037	0.796

DPC: decrease in platelet count; CI: confidence interval; BEV: balloon-expandable valve; EF: ejection fraction; AVA: aortic valve area

The univariate analyses revealed that a DPC of ≥ 50% was significantly associated with older age (OR: 1.088; *P *= 0.029), lower BMI (OR: 0.879; *P *= 0.016), and BEV use (unadjusted OR: 3.116; *P *= 0.019). The multivariable analysis revealed that a DPC of ≥ 50% was independently predicted by low BMI (adjusted OR: 0.884, 95% CI 0.785–0.997; *P *= 0.039) and BEV use (adjusted OR: 3.014, 95% CI 1.003–9.056; *P *= 0.045).

### Discussion

This study revealed that TAVI was strongly associated with a DPC, the nadir platelet count was reached on approximately postoperative day 3, and a large DPC was associated with BEV use and low BMI. Our findings regarding the nadir platelet count correspond with previous reports that the nadir platelet count is reached on day 2–3 and begins to recover on day 5 [4, 6–9].

It remains unclear whether post-TAVI thrombocytopenia differs according to the use of BEVs or SEVs [13]. Three studies and a sub-group analysis from a recent systematic review revealed that BEVs were associated with a higher risk of post-TAVI thrombocytopenia [7, 8, 11, 13]. Our study revealed similar results, although the underlying mechanisms remain unclear. The TAVI procedure may be associated with mechanical platelet destruction, increased coagulation, and inflammation-related platelet consumption [13], while other reports have suggested that thrombocytopenia is associated with BEV use because of endothelial damage and shear stress factors [7, 20–23]. Thus, it is possible that differences in prosthesis design and/or implantation technique generate variable degrees of endothelial damage and shear stress, which might underly the relationship between BEV use and thrombocytopenia [8, 21]. For example, BEV use might generate greater shear stress because of the balloon that is used during deployment. Hernandez-Enriquez et al. evaluated patients who received SEVs or BEVs and reported that the average DPC was 32.5 ± 13.9% [8], which is noticeably lower than the average DPC in our study (51 ± 13%). We suspect this difference is related to the greater proportion of BEV use in our study (84% vs. 57% of cases) [8]. Furthermore, Jilaihawi et al. [6] evaluated thrombocytopenia after only BEV implantation and reported that the average DPC was 61 ± 15%, which corresponds with our result.

Use of contrast agents may also influence the DPC [5, 10], which may be related to the agent’s chemical properties, immune-allergic reactions, or genetic predisposition [7, 8, 10, 23, 24]. For example, Mitrosz et al. reported that the DPC after TAVI using a BEV (Edwards Sapien XT) was associated with the contrast agent volume [5]. However, we did not observe any significant inter-group differences in the contrast agent volume, although we used smaller amounts (86.7 ± 26.0 mL) than those used by Mitrosz et al. (229.0 ± 74.3 mL) [5]. Our ability to use less contrast agent is likely related to the use of the transoesophageal echocardiography to confirm appropriate valve positioning.

A large DPC was associated with low BMI, which is also associated with mortality and thrombocytopenia risk after other high-risk percutaneous cardiac procedures [25, 26]. Moreover, patients with low BMI have increased risks of thrombocytopenia and acute myocardial infarction-related mortality, regardless of revascularisation status [26, 27]. Four reports have evaluated whether post-TAVI thrombocytopenia was associated with BMI [7, 8, 15, 16], although only Flaherty et al. reported a significant association [15]. Therefore, the relationship between post-TAVI thrombocytopenia and low BMI remains controversial, as previous reports have mainly evaluated European and American patients, who tend to have a higher BMI than Japanese patients [7, 8, 15, 16]. For example, the mean BMI in our study (22 kg/m^2^) was lower than the mean BMI in the aforementioned studies: 28.7 ± 4.7 kg/m^2^ [7], 26.0 ± 5.2 kg/m^2^ [8], 29 kg/m^2^ [15], and 26.9 kg/m^2^ [16]. To the best of our knowledge, this is the first report to examine whether low BMI was associated with post-TAVI DPC among Japanese patients, who are more likely to have a lower BMI and may be more prone to developing more severe thrombocytopenia. The relationship between thrombocytopenia and low BMI remains unclear; though large amounts of fluids are infused during the perioperative period in TAVI patients, and patients with a low BMI may be more susceptible to haemodilution, leading to a large DPC. Furthermore, we did not detect any instances of infection, sepsis, or disseminated intravascular coagulation leading to thrombocytopenia. Moreover, the mean intraoperative blood loss was 84.6 ± 115 mL, which suggests that thrombocytopenia was not likely related to platelet loss.

In conclusion, this study revealed that low BMI and BEV use were associated with a larger DPC after TAVI in Japanese patients with severe aortic valve stenosis. Platelet count monitoring after TAVI, especially when using BEV devices, is essential for Japanese patients, who are more likely to have a lower BMI.

## Limitations

This study has several limitations that should be acknowledged. First, the retrospective analysis of a small patient population is prone to bias related to unidentified confounders, and the results should be interpreted with caution. Second, use of PCPS was higher in our study compared to previous reports [28]. The reason for this is that prophylactic PCPS is used proactively for high risk cases of haemodynamic instability in our institution. We did not observe any significant inter-group differences in the use of PCPS, but the results should be interpreted with caution. Third, we cannot completely exclude the possibility of heparin-induced thrombocytopenia (HIT), as PF4 antibody detection was not performed as no patients had suspected HIT. Nevertheless, previous reports have suggested that HIT has little role in post-TAVI thrombocytopenia [4, 15, 16].

## Supplementary information

**Additional file 1: Figure S1.** Distributions of the baseline and nadir platelet counts among all patients

**Additional file 2: Figures S2.** Distributions of the times and nadir platelet counts among all patients

## Data Availability

The datasets used and/or analysed during the current study are available from the corresponding author on reasonable request.

## Abbreviations

BEV: Balloon-expandable valve
BMI: Body mass index
CI: Confidence interval
DPC: Decrease in platelet count
HIT: Heparin-induced thrombocytopenia
IQR: Interquartile range
OR: Odds ratio
PCPS: Percutaneous cardiopulmonary support
SEV: Self-expanding valve
TAVI: Transcatheter aortic valve implantation

## References

[1] Kodali SK, Williams MR, Smith CR, Svensson LG, Webb JG, Makkar RR (2012). Two-year outcomes after transcatheter or surgical aortic-valve replacement. N Engl J Med.

[2] Leon MB, Smith CR, Mack M, Miller DC, Moses JW, Svensson LG (2010). Transcatheter aortic-valve implantation for aortic stenosis in patients who cannot undergo surgery. N Engl J Med.

[3] Grube E, Laborde JC, Gerckens U, Felderhoff T, Sauren B, Buellesfeld L (2006). Percutaneous implantation of the CoreValve self-expanding valve prosthesis in high-risk patients with aortic valve disease: the Siegburg first-in-man study. Circulation.

[4] Dvir D, Genereux P, Barbash IM, Kodali S, Ben-Dor I, Williams M (2014). Acquired thrombocytopenia after transcatheter aortic valve replacement: clinical correlates and association with outcomes. Eur Heart J.

[5] Mitrosz M, Kazimierczyk R, Sobkowics B, Waszkiewicz E, Kralisz P, Frank M (2017). The causes of thrombocytopenia after transcatheter aortic valve implantation. Thromb Res.

[6] Jilaihawi H, Doctor N, Chakravarty T, Kashif M, Mirocha J, Cheng W (2015). Major thrombocytopenia after balloon-expandable transcatheter aortic valve replacement: prognostic implications and comparison to surgical aortic valve replacement. Catheter Cardiovasc Interv..

[7] Hernandez-Enriquez M, Regueiro A, Romaguera R, Andrea R, Gómez-Hospital JA, Pujol-López M (2019). Thrombocytopenia after transcatheter aortic valve implantation. A comparison between balloon-expandable and self-expanding valves. Catheter Cardiovasc Interv..

[8] Hernandez-Enriquez M, Chollet T, Bataille V, Campelo-Parada F, Boudou N, Bouisset F (2019). Comparison of the frequency of thrombocytopenia after transfemoral transcatheter aortic valve implantation between balloon-expandable and self-expanding valves. Am J Cardiol.

[9] Mitrosz M, Chlabicz M, Hapaniuk K, Kaminski KA, Sobkowicz B, Piszcz J (2017). Thrombocytopenia associated with TAVI—The summary of possible causes. Adv Med Sci..

[10] Gallet M, Seemann A, Yamamoto M, Hayat D, Mouillet G, Monin JL (2013). Effect of transcatheter (via femoral artery) aortic valve implantation on the platelet count and its consequences. Am J Cardiol.

[11] Jiritano F, Santarpino G, Serraino GF, Ten Cate H, Matteucci M, Fina D (2019). Peri-procedural thrombocytopenia after aortic bioprosthesis implant: a systematic review and meta-analysis comparison among conventional, stentless, rapid-deployment, and transcatheter valves. Int J Cardiol.

[12] Motroz M, Kazimierczyk R, Chlabicz M, Sobkowicz B, Waszkiewicz E, Lisowska A (2018). Perioperative thrombocytopenia predicts poor outcome in patients undergoing transcatheter aortic valve implantation. Adv Med Sci..

[13] Takahashi S, Yokoyama N, Watanabe Y, Katayama T, Hioki H, Yamamoto H (2020). Predictor and mid-term outcome of clinically significant thrombocytopenia after transcatheter aortic valve selection. Circ J.

[14] Sedaghat A, Falkenberg N, Sinning JM, Kulka H, Hammerstingl C, Nickenig G (2016). TAVI induces an elevation of hemostasis-related biomarkers, which is not causative for post-TAVI thrombocytopenia. Int J Cardiol.

[15] Flaherty MP, Mohsen A, Moore JB, Bartoli CR, Schneibel E, Rawasia W (2015). Predictors and clinical impact of pre-existing and acquired thrombocytopenia following transcatheter aortic valve replacement. Catheter Cardiovasc Interv..

[16] McCabe JM, Huang PH, Riedl LA, Devireddy SR, Grondell J, Connors AC (2014). Incidence and implications of idiopathic thrombocytopenia following transcatheter aortic valve replacement with the Edwards Sapien(©) valves: a single center experience. Catheter Cardiovasc Interv..

[17] Özen MB, Ayhan H, Kasapkara HA, Keleş T, Durmaz T, Erdoğan KE (2017). The effect of transcatheter aortic valve implantation on mean platelet volume. Turk J Med Sci..

[18] Gul M, Uyarel H, Akgul O, Uslu N, Yildirim A, Eksik A (2014). Hematologic and clinical parameters after transcatheter aortic valve implantation (TAVI) in patients with severe aortic stenosis. Clin Appl Thromb Hemost.

[19] Kappetein AP, Head SJ, Genereux P, Piazza N, van Mieghem NM, Blackstone EH (2012). Updated standardized endpoint definitions for transcatheter aortic valve implantation: the Valve Academic Research Consortium-2 consensus document. Eur Heart J.

[20] Miceli A (2016). Tissue valve, nitinol stent, or storage solution? The mystery still goes on. J Thoracic Cardiovasc Surg..

[21] Nobili M, Sheriff J, Morbiducci U, Redaelli A, Bluestein D (2008). Platelet activation due to hemodynamic shear stresses: damage accumulation model and comparison to in vitro measurements. ASAIO J.

[22] Jiritano F, Cristodoro L, Malta E, Mastroroberto P (2016). Thrombocytopenia after sutureless aortic valve implantation: comparison between Intuity and Perceval bioprostheses. J Thorac Cardiovasc Surg.

[23] Miceli A (2020). Commentary: thrombocytopenia yes…thrombocytopenia no…that is the question. J Thorac Cardiovasc Surg.

[24] De Labriolle A, Bonello L, Lemesle G, Roy P, Steinberg DH, Xue Z (2010). Decline in platelet count in patients treated by percutaneous coronary intervention: definition, incidence, prognostic importance, and predictive factors. Eur Heart J.

[25] Brunner MP, Cronin EM, Duarte VE, Yu C, Tarakji KG, Martin DO (2014). Clinical predictors of adverse patient outcomes in an experience of more than 5000 chronic endovascular pacemaker and defibrillator lead extractions. Heart Rhythm..

[26] Nikolsky E, Stone GW, Grines CL, Cox DA, Garcia E, Tcheng JE (2006). Impact of body mass index on outcomes after primary angioplasty in acute myocardial infarction. Am Heart J.

[27] Wang TY, Ou FS, Roe MT, Harrington RA, Ohman EM, Gibler WB (2009). Incidence and prognostic significance of thrombocytopenia developed during acute coronary syndrome in contemporary clinical practice. Circulation.

[28] Raffa G, Kowalewski M, Meani P, Follis F, Martucci G, Arcadipane A (2019). In-hospital outcomes after emergency or prophylactic veno-arterial extracorporeal membrane oxygenation during transcatheter aortic valve implantation: a comprehensive review of the literature. Perfusion..

