# Supplementary material for: Predictors of short-term thrombocytopenia after transcatheter aortic valve implantation: a retrospective study at a single Japanese center
Source: BMC Res Notes. 2020 Nov 16;13:536. doi: 10.1186/s13104-020-05386-7 (PMC7670721; doi:10.1186/s13104-020-05386-7)
Supplement: Supplementary file 1 — Additional file 1: Figure S1. Distributions of the baseline and nadir platelet counts among all patients [file 13104_2020_5386_MOESM1_ESM.pptx]

## Slide 1
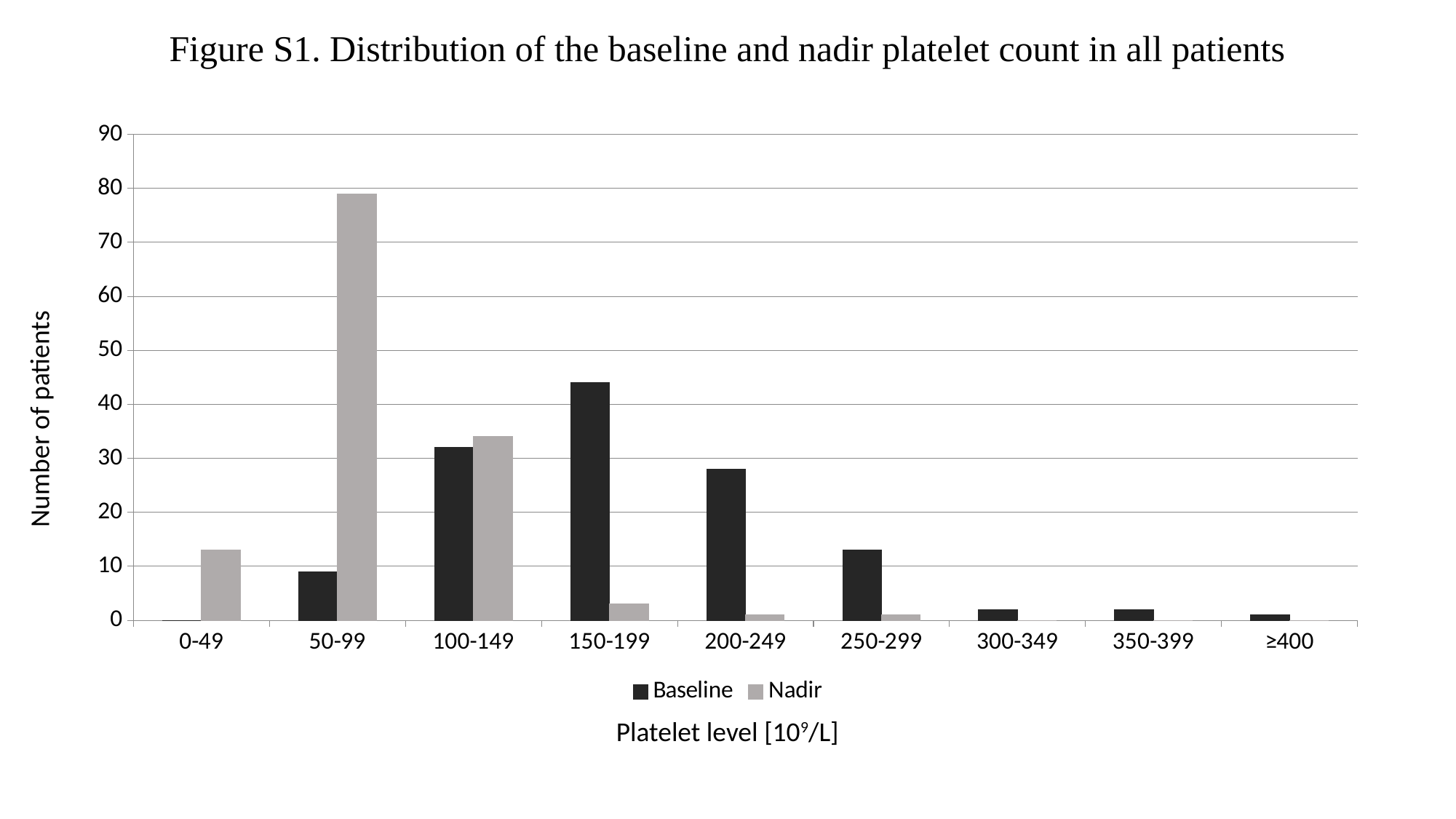

Figure S1. Distribution of the baseline and nadir platelet count in all patients
### Chart
| Category | Baseline | Nadir |
|---|---|---|
| 0-49 | 0.0 | 13.0 |
| 50-99 | 9.0 | 79.0 |
| 100-149 | 32.0 | 34.0 |
| 150-199 | 44.0 | 3.0 |
| 200-249 | 28.0 | 1.0 |
| 250-299 | 13.0 | 1.0 |
| 300-349 | 2.0 | 0.0 |
| 350-399 | 2.0 | 0.0 |
| ≥400 | 1.0 | 0.0 |Number of patients
Platelet level [109/L]
