# Supplementary material for: Predictors of short-term thrombocytopenia after transcatheter aortic valve implantation: a retrospective study at a single Japanese center
Source: BMC Res Notes. 2020 Nov 16;13:536. doi: 10.1186/s13104-020-05386-7 (PMC7670721; doi:10.1186/s13104-020-05386-7)
Supplement: Supplementary file 2 — Additional file 2: Figures S2. Distributions of the times and nadir platelet counts among all patients [file 13104_2020_5386_MOESM2_ESM.pptx]

## Slide 1
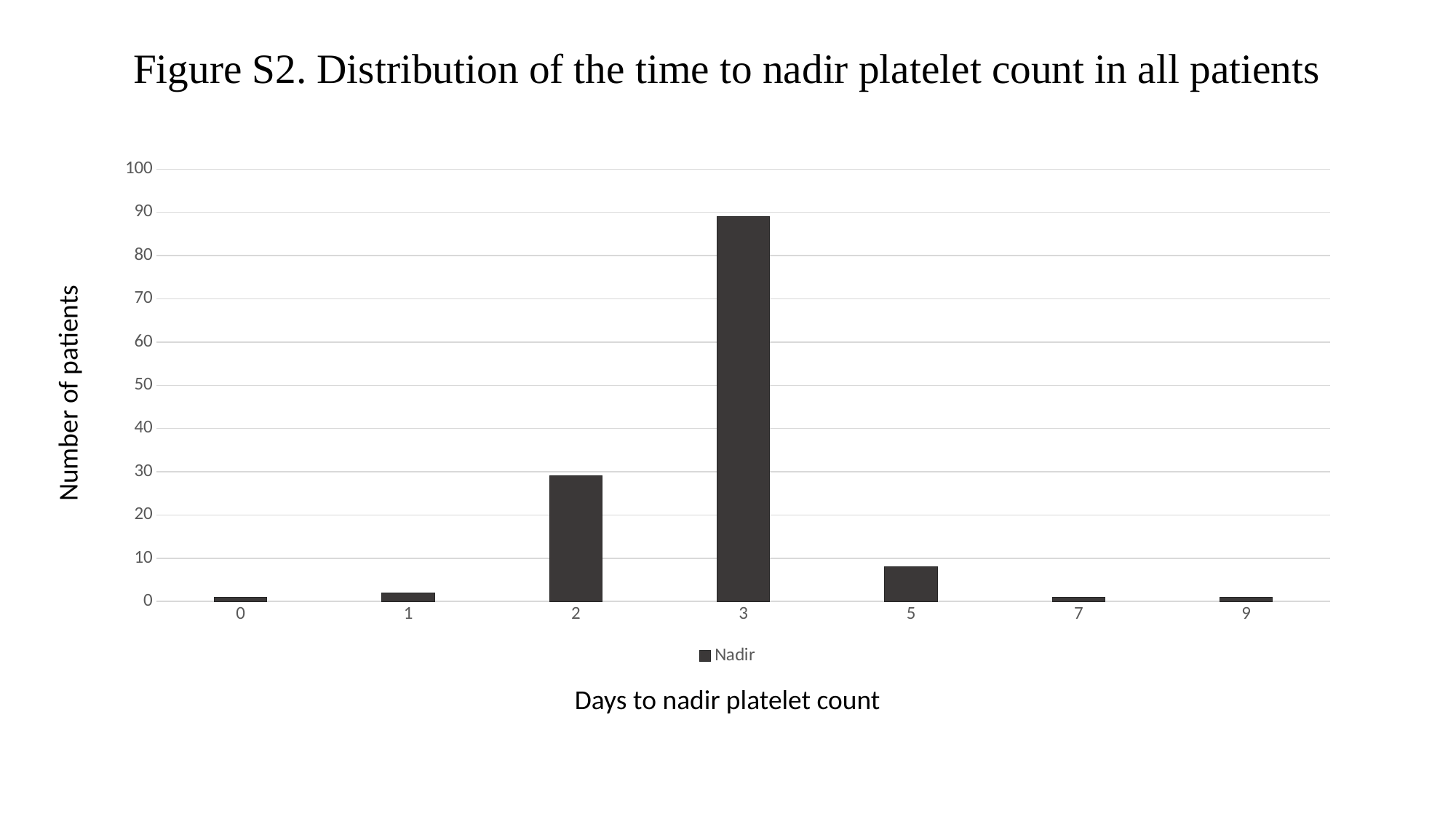

Figure S2. Distribution of the time to nadir platelet count in all patients
### Chart
| Category | Nadir |
|---|---|
| 0 | 1.0 |
| 1 | 2.0 |
| 2 | 29.0 |
| 3 | 89.0 |
| 5 | 8.0 |
| 7 | 1.0 |
| 9 | 1.0 |Number of patients
Days to nadir platelet count
